# Imaging in metastatic breast cancer, CT, PET/CT, MRI, WB-DWI, CCA: review and new perspectives

**DOI:** 10.1186/s40644-023-00557-8

**Published:** 2023-05-31

**Authors:** Dione Lother, Marie Robert, Elliot Elwood, Sam Smith, Nina Tunariu, Stephen R.D. Johnston, Marina Parton, Basrull Bhaludin, Thomas Millard, Kate Downey, Bhupinder Sharma

**Affiliations:** 1 The Royal Marsden Hospital, London & Sutton, UK; 2 Institut de Cancérologie de l’Ouest, St Herblain, France; 3 The Institute of Cancer Research (ICR), London & Sutton, UK

**Keywords:** Metastatic breast cancer, Staging, Response assessment, Multimodal, Multiparametric, Anatomo-functional imaging

## Abstract

**Background:**

Breast cancer is the most frequent cancer in women and remains the second leading cause of death in Western countries. It represents a heterogeneous group of diseases with diverse tumoral behaviour, treatment responsiveness and prognosis. While major progress in diagnosis and treatment has resulted in a decline in breast cancer-related mortality, some patients will relapse and prognosis in this cohort of patients remains poor. Treatment is determined according to tumor subtype; primarily hormone receptor status and HER2 expression. Menopausal status and site of disease relapse are also important considerations in treatment protocols.

**Main body:**

Staging and repeated evaluation of patients with metastatic breast cancer are central to the accurate assessment of disease extent at diagnosis and during treatment; guiding ongoing clinical management. Advances have been made in the diagnostic and therapeutic fields, particularly with new targeted therapies. In parallel, oncological imaging has evolved exponentially with the development of functional and anatomical imaging techniques. Consistent, reproducible and validated methods of assessing response to therapy is critical in effectively managing patients with metastatic breast cancer.

**Conclusion:**

Major progress has been made in oncological imaging over the last few decades. Accurate disease assessment at diagnosis and during treatment is important in the management of metastatic breast cancer. CT (and BS if appropriate) is generally widely available, relatively cheap and sufficient in many cases. However, several additional imaging modalities are emerging and can be used as adjuncts, particularly in pregnancy or other diagnostically challenging cases. Nevertheless, no single imaging technique is without limitation. The authors have evaluated the vast array of imaging techniques – individual, combined parametric and multimodal - that are available or that are emerging in the management of metastatic breast cancer. This includes WB DW-MRI, CCA, novel PET breast cancer-epitope specific radiotracers and radiogenomics.

**Supplementary Information:**

The online version contains supplementary material available at 10.1186/s40644-023-00557-8.

## Background

Breast cancer is the most frequent cancer in women and the second leading cause of death in Western countries [1]. It represents a heterogeneous group of diseases with diverse tumoral behaviour, treatment responsiveness and prognosis [2]. Despite a decline in breast cancer-related mortality, prognosis in advanced disease remains poor. After 5 years of adjuvant endocrine therapy, there is a 10–41% risk of distant recurrence depending on stage and tumor grade [3]. At presentation, approximately 4–10% of breast cancers are metastatic and accurate staging is therefore essential in guiding management and optimizing overall patient outcome [4]. Treatment is determined according to tumor subtype; primarily hormone receptor status and HER2 expression. Menopausal status and site of disease relapse are also important considerations in treatment protocols [1]. Major advances have been made in the diagnostic and therapeutic fields, particularly with new targeted therapies. In parallel, oncological imaging has evolved exponentially with the development of functional and anatomical imaging techniques. Consistent, reproducible and validated methods of assessing response to therapy are critical in effectively managing patients with metastatic breast cancer.

In this review, we outline staging guidelines and consider the strength and limitations of current clinical practice within the context of international mandates for evidence-based medicine, cost-effective clinical practice and patient safety. The keywords “metastatic breast cancer”, "staging", "response assessment", "multimodal", "multiparametric", "anatomo-functional" and "radiogenomics" were applied in a systematic search using the online database Pubmed conducted between July 2021 and March 2023. Original manuscripts, systematic reviews and international guidelines published in peer-reviewed and indexed journals between January 1995 and March 2023 were considered. The publication relevance was determined manually by two independent authors who then extracted the study details and relevant data. We describe the evolution of diagnostic technology and evaluate the applications of established anatomo-functional, as well as novel molecular and radiomic-based imaging techniques in the context of metastatic breast cancer.

## Current practice in disease assessment and follow-up for metastatic breast cancer

**Fig. 1:**
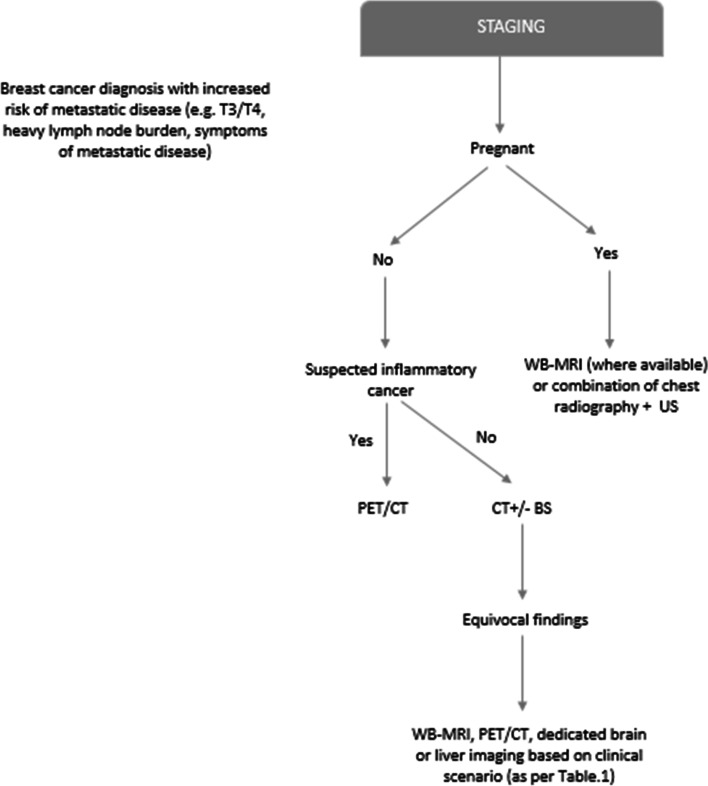
Suggested decision-tree/diagnostic schema for initial staging (if appropriate)

Breast cancer diagnosis is based on histopathological assessment of the primary tumor or metastases according to the American Joint Committee on Cancer (AJCC) TMN system [5]; whereas staging - evaluating the extent of visceral, nodal and bone disease, is determined largely on imaging. Due to the heterogeneity of breast cancer, consensus on the optimal imaging modality or interval frequency is however currently lacking. Initial staging and restaging imaging protocols are based upon both national and international guidelines, which are varied. The Royal College of Radiologists (RCR) clinical practice guidelines for breast cancer do not advocate routine staging imaging for asymptomatic patients with early stage (T1/T2) disease, rather imaging is usually reserved for those patients with more advanced cancers at higher risk of metastasis (T3/T4)^6^. RCR guidelines further recommend computerised tomography (CT) of the thorax, abdomen and pelvis with or without bone scintigraphy (BS) for staging of patients with large (T4) tumors, heavy burden of nodal disease (N2/N3)^6^ or symptoms attributable to metastatic disease. Positron emission tomography fused with CT (PET/CT) is recommended in cases of suspected inflammatory breast cancer [6]. This is supported by van Uden et al. in their recent systematic review demonstrating that 2-deoxy-2 [^18^ F] fluoro-D-glucose PET/CT (FDG-PET/CT) outperforms conventional imaging in the detection of locoregional and distant metastases in the initial diagnostic workup of locally advanced and inflammatory breast cancers [7]. The North American National Comprehensive Cancer Network (NCCN) recommends CT and BS to assess metastatic disease primarily [8]. National Institute for Health and Care Excellence (NICE) guidelines recommend CT, magnetic resonance imaging (MRI), ultrasound and plain radiography to assess the extent of visceral disease. For bone disease, BS, CT or MRI is recommended [9]. The NCCN also advise that FDG-PET/CT in this setting should be employed only when conventional image findings are inconclusive or suspicious (Fig. 1). Current evidence does not support the routine use of FDG-PET/CT in the staging of locoregional disease. The European Society for Medical Oncology (ESMO) guidelines recommend clinical history, physical examination, hematology and biochemistry tests together with imaging of the skeleton, chest and abdomen as the minimal staging work-up in patients at high risk of developing metastatic disease (i.e. those with heavy disease burden or aggressive tumoral biology) [10]. Other options include ultrasound, particularly in resource-poor countries. Further, the ESMO recommends the application of validated gene expression profiles as complement to other staging tools, where these may assist with prognostication and clinical management. Staging and risk assessment recommendations outlined by the ESMO have in addition been agreed and accepted by the Pan-Asian ESMO adapted Clinical Practice guidelines [11]. In cases where staging imaging is indicated, there is consensus that the initial minimum imaging work-up should include CT evaluation of the thorax and abdomen as well as BS [12]. Conversely, CT evaluation of the pelvis is not routinely indicated. In a study of 2426 women with metastatic breast cancer, pelvic metastases were the only known site of disease in 0.5% (*n* = 13) of cases, of which the majority were osseous in origin. Pelvic CT led to 204 additional imaging procedures and 50 surgical procedures of which 84.6% yielded normal, benign or indeterminate results [13]. Where there is concerning neurological symptoms indicative of intracranial or spinal disease, brain and spine MRI are also recommended.

Regarding response assessment, the ESMO guidelines suggest repeating initial imaging of target lesions every 8–16 weeks in patients treated with endocrine therapy and every 2–4 cycles for those treated with chemotherapy [10]. The NCCN recommends that interval frequency of CT and BS should be determined based on specific treatment type (endocrine therapy versus chemotherapy).

**Fig. 2:**
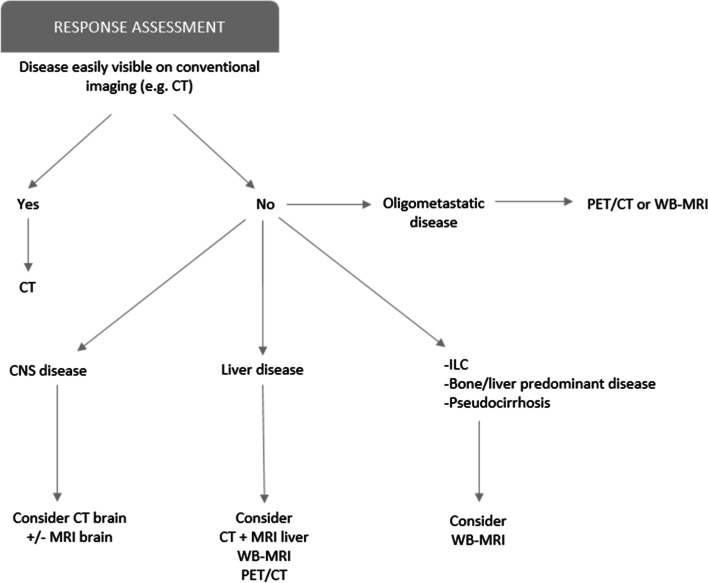
Suggested decision-tree /diagnostic schema for assessing treatment response (restaging)

Various imaging modalities can be used to assess the extent of disease as well as response to treatment. In standard clinical practice, CT is the most widely used (Fig. 2). Many guidelines recommend the use of Response Evaluation Criteria In Solid Tumors (RECIST 1.1) in CT reporting to establish whether there is complete response, partial response, stable disease or progressive disease [9].

BS with technicium-99 m methylene diphosphonate (99mTc-MDP) remains the mainstay of osseous metastatic disease evaluation [14]. The performance of BS is improved with its modern extension: BS with Single Photon Emission Computed Tomography (SPECT) and CT-SPECT [15]. However, NICE guidelines and the RECIST Working Group state “there is no evidence that BS can be used to assess response to treatment” primarily due to its inability to differentiate between osteoblastic response and disease progession [9, 16].

FDG-PET/CT is a functional imaging modality which displays changes in metabolic activity over time, measuring glucose uptake and retention within tumors. This imaging technique has the potential advantage of detecting metabolic activity prior to changes in anatomical morphology. Although there is very little published data demonstrating a clear overall survival benefit of FDG-PET/CT over and above conventional imaging modalities in the context of staging [10], FDG-PET/CT has been shown to detect unsuspected extra-axillary nodal and distant metastases particularly in stage III disease and can potentially be utilised in this scenario [17].

With the mounting pressures on most health care systems, healthcare policy makers are focusing attention on value-based health care that continues to promote quality and improve patient outcomes, while limiting the overuse of advanced imaging techniques. Accordingly, imaging departments are adapting by developing high speed imaging protocols [18]. In centres where there is good physics support, a whole-body MRI (WB-MRI) can now be performed in 30–40 min which includes diffusion-weighted imaging (DWI), T2-weighted and T1-weighted DIXON sequences [19] (further discussed in "Novel drugs and response-assessment techniques" section). There has also been development of organ or region-specific fast protocols, for example in the liver [20].

Digitally supported techniques, such as machine learning and other artificial intelligence (AI) techniques are being developed to support imaging. The combination of whole-body diffusion-weighted MRI (WB DWI-MRI) and machine learning has been used in the detection and evaluation of disease extent before and after systemic treatment for example [21] (further discussed in "Radiogenomics in metastatic breast cancer" section ).

Novel molecular technologies used in conjunction with MRI are also gaining attention in the sphere of oncological imaging.

Tumour or organ-specific contrast agents, such as those based on iron oxide and dendrimer nanomaterials are being evaluated and can afford better characterisation of metastases in some settings. Ultra-small superparamagnetic iron oxide (USPIO) compounds are an example, with several different preparations already approved by the US Food and Drug Administration (FDA) and available in clinical practice in the USA. Harada et al. assessed the utility of USPIO enhanced MRI in the detection of axillary lymph node metastases in breast cancer and reported a sensitivity, specificity and overall accuracy of 86.4%, 97.5% and 95%, respectively on post USPIO enhanced MRI compared with a sensitivity, specificity and overall accuracy of 36.5%, 94.1% and 81.0%, respectively on conventional MRI [22].

## Imaging: pitfalls and risks

### Bone disease assessment

**Fig. 3:**
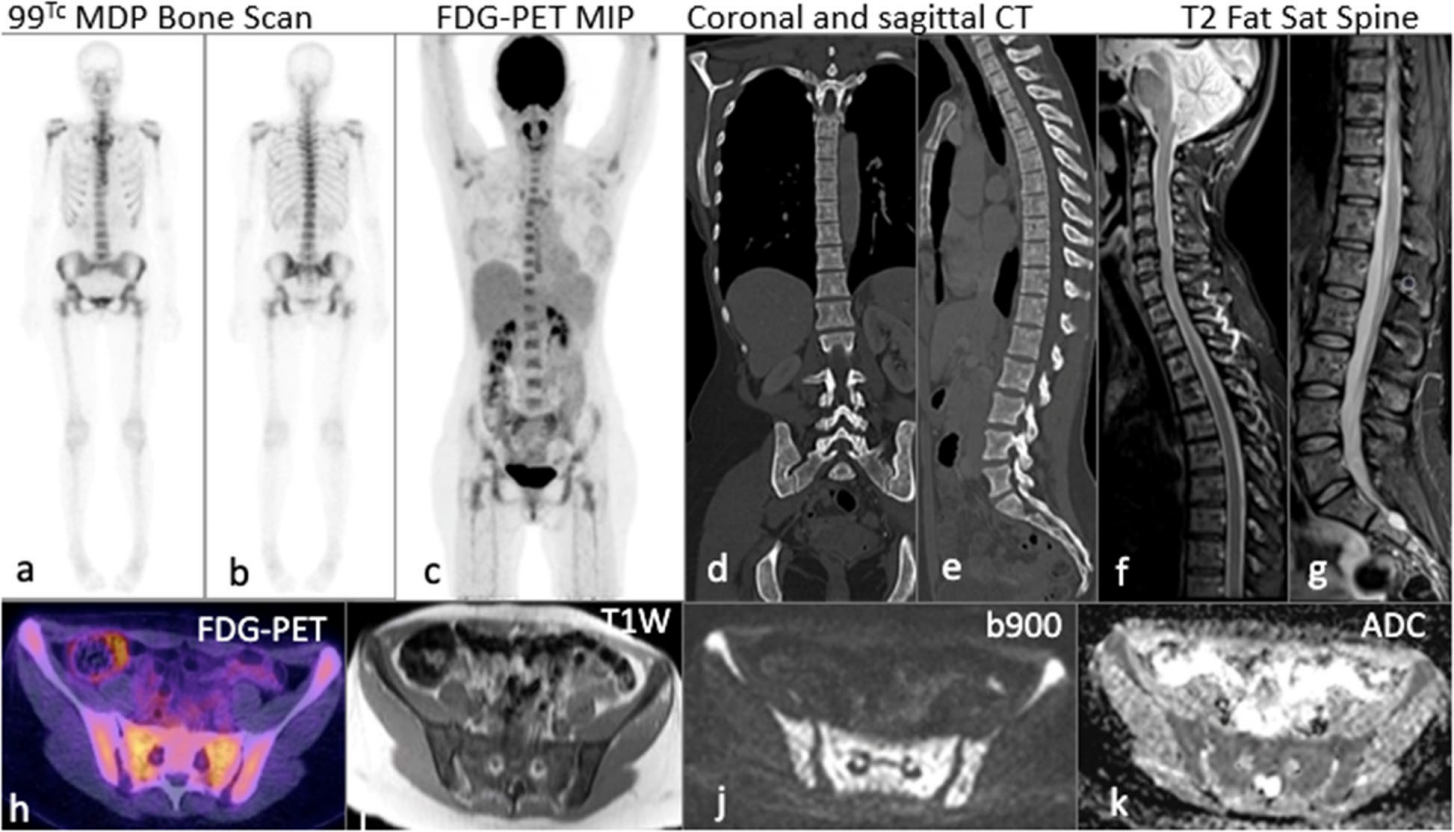
45-year-old female with multifocal grade 2 invasive ER positive HER2 negative metastatic lobular breast cancer: 99m Tc MDP planar bone scan (**a**, **b**) and coronal maximum intensity projection PET/CT (**c**) show no uptake in the axial or appendicular skeleton. Coronal and sagittal CT reformats (**d**, **e**) performed 1 week later demonstrating subtle sclerotic changes. Sagittal fat saturated T2W sequence of the spine (**f**, **g**) shows heterogenous marrow signal. Fused axial PET/CT (**h**) shows no FDG avidity. Widespread bone metastases in the same patient on WB DW-MRI (**i**) axial T1W (**j**) b900 and (**k**) ADC

BS remains central in metastatic osseous disease evaluation. Planar BS is useful in identifying metastatic bone disease as it is reasonably sensitive [23], with 99mTc-MDP binding to bone as a result of osteoblastic activity [24]. However, BS lacks specificity, anatomical detail [25] and does not always reflect the true extent of disease within the bone marrow (Fig. 3). Furthermore, well described ‘flare reactions’ with temporary increase in activity on BS, can be seen in patients who later respond to therapy [26] (Table 1). Flare may therefore be incorrectly interpreted as disease progression and lead to an inappropriate modification in treatment regime [27]. Coleman et al. studied changes in osteoblastic function in 53 patients treated with systemic therapy for bone metastases in advanced breast cancer. In 12 of 16 patients with documented healing lytic disease on plain radiography, increased activity in baseline lesions with new foci of tracer uptake was incorrectly interpreted as disease progression on BS after 3 months of systemic treatment [28]. Indeed, with treatment response, there is an osteoblastic reaction which provokes an increase in 99mTc-MDP uptake similar to that seen in disease progression which can be misinterpreted as treatment failure. For this reason, repeat BS at a later date is recommended when new lesions will serve as a more accurate indicator of disease progression. However, in rapidly progressive disease, new bone formation is limited and a decrease in uptake has been described. Moreover, in patients with ‘superscans’ (very advanced disease in bones), response cannot be objectively assessed as new lesions may not be detected on a background of already elevated 99mTc-MDP uptake [29]. Therefore, NICE and the ESMO advise against the use of BS for response assessment of bone metastases [9, 10, 12].

**Table 1:** Optimal/favored imaging modality/sequence based on clinical question

Clinical question	Optimal imaging modality/sequence	Considerations
Presence of osteoblastic metastases	99mTc-MDP	Poor specificity, afftected by ‘flare’ reactions
Presence of osteolytic or osteoblastic metastases, active disease residuum versus treatment response	FDG-PET/CT WB-MRI	WB-MRI not widely available as yet, longer image acquisition times and requirement to train radiologists in interpretation
Presence of parenchymal CNS metastases	Contrast-enhanced T1 MRI	Higher sensitivity in detection of parenchymal versus leptomeningeal disease
Presence of leptomenigeal disease	Contrast-enhanced FLAIR MRI	Limited, small-scale studies
Residual CNS disease versus treatment-related effects	CCA	Inherently lengthy image acquisition times, require dedicated neuroradiology interpretation
Presence of hepatic metastases	CT LIVER MRI WB-MRI	Differentiating active disease residuum from pseudo-cirrhosis of malignancy often challenging on CT
Residual hepatic disease versus pseudo-cirrhosis of malignancy	WB-MRI	Interpretation influenced by radiologist experience
Presence of oligometastatic disease	FDG-PET/CT WB-MRI	WB-MRI less widely available than FDG-PET/CT
Presence of peritoneal carcinomatosis	WB-MRI	WB-MRI not widely available as yet as above

CT performance is poor when it comes to osseous disease [30]. In particular, it is recognised that CT is inferior to WB-MRI and FDG-PET/CT in the evaluation of bone metastases [14, 31]. Furthermore, according to RECIST 1.1 criteria, sclerotic metastases are considered ‘unmeasurable lesions’ [12]. Conversely, lytic metastases with a soft tissue component of ≥ 10 mm are measurable in a similar manner to that of other soft tissue lesions.

FDG-PET/CT is more specific than BS as it detects active tumor cells in the skeleton. Moreover, lytic bone metastases are more easily detected with FDG-PET/CT (Table 1). The current sensitivity of FDG-PET/CT in lytic bone disease detection is 85% compared with 75% for BS. In a study including 23 breast cancer patients with known bone metastases, FDG-PET/CT detected more sites of disease than BS (mean 14.1 versus 7.8 lesions, respectively) [31]. The reported difference was more important with osteolytic disease. In a large retrospective study conducted by the author’s group at the Royal Marsden Hospital over a 4-year period, 233 FDG-PET/CT studies were performed in 122 breast cancer patients, 72% of which had recurrent or metastatic disease. Concordance between BS and FDG-PET/CT was reported in 70% of cases. In the remaining 30%, FDG-PET/CT identified lytic bone lesions not detected on BS [32]. However, the effective whole body radiation dose resulting from FDG-PET/CT is significantly higher than that of BS, ranging from 20 to 25 mSv [33] compared with 2.9-5 mSv [34].

Although CT and BS are inferior to WB-MRI and FDG-PET/CT in the evaluation of bone metastases, they remain in wide use in the assessment of metastatic breast cancer as these examinations are more readily available at most institutions and can be undertaken quickly with a lower cost. However, although WB-MRI and FDG-PET/CT are more expensive, the treatment of complications from bone metastases can be associated with high costs, such as inpatient admissions, imaging and treatment related to pathological bone fractures and cord compression [14]. Earlier identification of bone metastases with WB-MRI and FDG-PET/CT could reduce the incidence of such complications. However, at present there is lack of data relating to the cost-effectiveness of these different imaging modalities [35]. The ESMO guidelines conclude that “the role of FDG-PET/CT in monitoring bone response to therapy has been reported in few small studies and appears potentially promising; however, prospective trials are needed to establish its true clinical utility” [10].

### Craniospinal disease assessment

Disease spread to the central nervous system (CNS) is considered a late complication of metastatic breast cancer, with 10–16% of patients with stage IV disease at presentation going on to develop metastases to the brain parenchyma, spinal cord or leptomeninges [36]. While, routine neuroimaging is not recommended in asymptomatic patients [9, 12], evaluation of the entire neuroaxis, including the brain and spine is indicated in cases where CNS metastases are suspected. Contrast-enhanced CT and MRI have been the mainstay of imaging in the diagnostic evaluation of CNS metastases in symptomatic patients with advanced metastatic disease. Gadolinum-enhanced T1-weighted MRI has been shown to have superior sensitivity to that of contrast-enhanced CT [37] and unenhanced Fluid-Attenuated Inversion Recovery (FLAIR) MRI in the diagnosis of leptomeningeal disease [38]. More recently, contrast-enhanced FLAIR imaging has shown promise and high sensitivity in the detection of subarachnoid disease [38]. Given the associations of Gadolinium-based contrast agent exposure and nephrogenic systemic fibrosis and Gadolinium deposition disease, use of Gadolinium-enhanced MRI, especially in high-risk groups (e.g. pregnancy, renal impairment), or in the context of repeated examinations requires particular caution [39]. FDG-PET/CT has a low sensitivity for the detection of leptomeningeal disease and currently has little role in the diagnosis of CNS metastases [40].

### Hepatic disease assessment

CT is a useful imaging modality in the evaluation of metastatic liver disease. RECIST 1.1 measurements have been found to be reproducible with a large and heterogeneous population of radiologists. Given that CT is still more widely available than MRI, this modality is often the first line staging tool in the assessment of liver metastases. CT assessment of hepatic disease and particularly residual disease, can however be challenging [41]. Moreover, approximately 10–26% of liver metastases of breast origin are hyperenhancing, with 4–17% showing mixed vascularity [42]. Such hyperenhancing metastases demonstrate variable characteristics; up to 59% can be isodense or hypodense to liver parenchyma on either portal venous or arterial phase imaging. As such, it has been argued that multiphase imaging is often required to accurately detect metastases to the liver [42]. However, in a recent retrospective study of 7621 newly diagnosed breast cancer cancers, Ko et al. found no statistical difference in MRI referral rate, negative MR rate, true positive CT rate and overall liver metastasis rate between patients undergoing single and multiphase liver CT [43]. Furthermore, multiphase liver CT delivers a significantly higher radiation dose than single-phase studies (median effective dose of 31 mSv - equivalent to 442 chest radiographs or 15 years of background radiation exposure) [44], and thus should be used with caution.

**Fig. 4:**
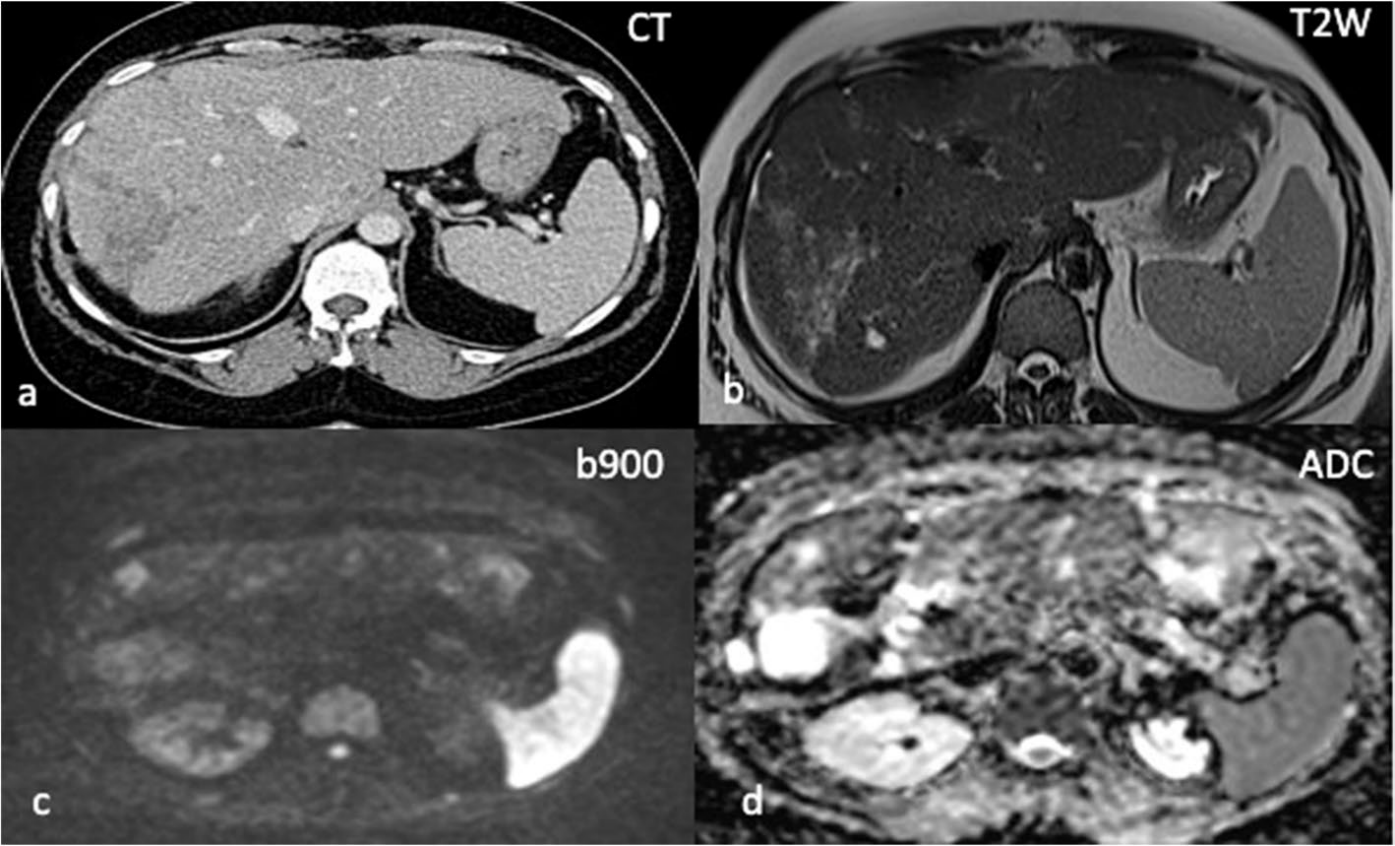
Pseudocirrhosis of the liver in 59-year-old female with relapsed grade 3 ER positive HER2 negative metastatic invasive ductal carcinoma of the left breast. Axial CT performed in May 2020 demonstrates a nodular hepatic contour with capsular retraction (a). Increasing hypodensity within the right lobe was felt to represent disease progression. WB DWI-MRI performed 3 weeks later in June shows high b900 signal and corresponding increased ADC values (**c**, **d**) at sites of previously identified disease in the right lobe of liver in keeping with maintained treatment response

Another diagnostic challenge is the concept of *‘pseudo-cirrhosis of malignancy’* where the liver develops a fibrotic appearance with nodularity and capsular retraction in response to chemotherapy [45, 46]. In such cases, distinguishing active liver disease from post-treatment fibrotic change is often very difficult (Fig. 4), which can be particularly significant in cases with previous extensive disease infiltration of the liver [47]. FDG-PET/CT is a useful adjunct here, where a negative study is seen in the context of a controlled fibrotic liver and *‘active macroscopic’* metastatic liver disease may be detected [48]. MRI is however superior to ultrasound, CT and FDG-PET/CT in terms of contrast resolution and has the ability to provide both morphological and physiological information and is therefore the modality of choice for the assessment of liver disease [47, 48]. Small hepatic metastases are often detectable on high b-value DWI before they are appreciable on other sequences [48]. Thin slice MRI with DWI is the best imaging modality to detect liver metastases.

### Oligometastatic disease

Approximately 1–10% of breast cancer patients will have oligometastatic disease [49]. Oligometastatic disease (OMD) is a distinct subset of metastatic cancer and has been defined as the presence of 1 to 5 metastases in less than 2 organs, although the exact number of metastatic sites is debated [50]. Detection of patients with OMD, who may be suitable for metastatic-directed therapies offering curative intent is crucially important. Various authors have shown benefits in local control, progression-free survival (PFS) and overall survival (OS) in patients with oligometastatic breast cancer treated with local metastatic-directed therapies. Milano et al. reported 2-year and 4-year OS rates of 74% and 59%, respectively in patients with oligometastatic breast cancer who underwent stereotactic ablative body radiation [51]. Similarly, Lee et al. reported prospective data of stereotactic ablative body radiation in patients with oligometastatic cancer, including those with breast cancer [52]. The group reported local control rates of lung metastases of 100% and 90% at 1 and 2 years, respectively. To date, FDG-PET/CT is the most accessible and sensitive diagnostic imaging modality in this context (sensitivity of 90–94% and accuracy rate of 83–90%) [53]. Although WB-MRI is also very sensitive, its specificity is poor in detecting locoregional and metastatic disease (82% false positive rate compared to 11% on FDG-PET/CT) [54].

### Specific breast cancer subtype: lobular breast cancer-peritoneal carcinomatosis

Metastatic invasive lobular carcinoma (ILC) poses a diagnostic challenge to radiologists due to its often infiltrative pattern of spread to serosal surfaces, the retroperitoneum and gastrointestinal/genitourinary tracts [55]. In addition, a proportion of ILC will be hypometabolic and as such, detection on conventional and functional imaging is notoriously difficult. As a result, ILC metastases commonly present late with secondary sequelae (e.g. hydronephrosis, bowel obstruction or liver failure) [56]. Regarding peritoneal carcinomatosis, CT is the most common imaging modality employed to assess the peritoneum but requires intravenous contrast. Furthermore, its suboptimal contrast resolution reduces its ability to detect small peritoneal implants. For example, sensitivity has been reported to be as low as 25% for implants of less than 0.5 cm compared with 90% for those of more than 5 cm [57]. Conversely, MRI has been proven to be more accurate in detecting small peritoneal deposits and carcinomatosis due to its superior soft tissue contrast and ability to provide additional information about tissue characteristics with the addition of dynamic contrast-enhanced imaging [58].

**Fig. 5:**
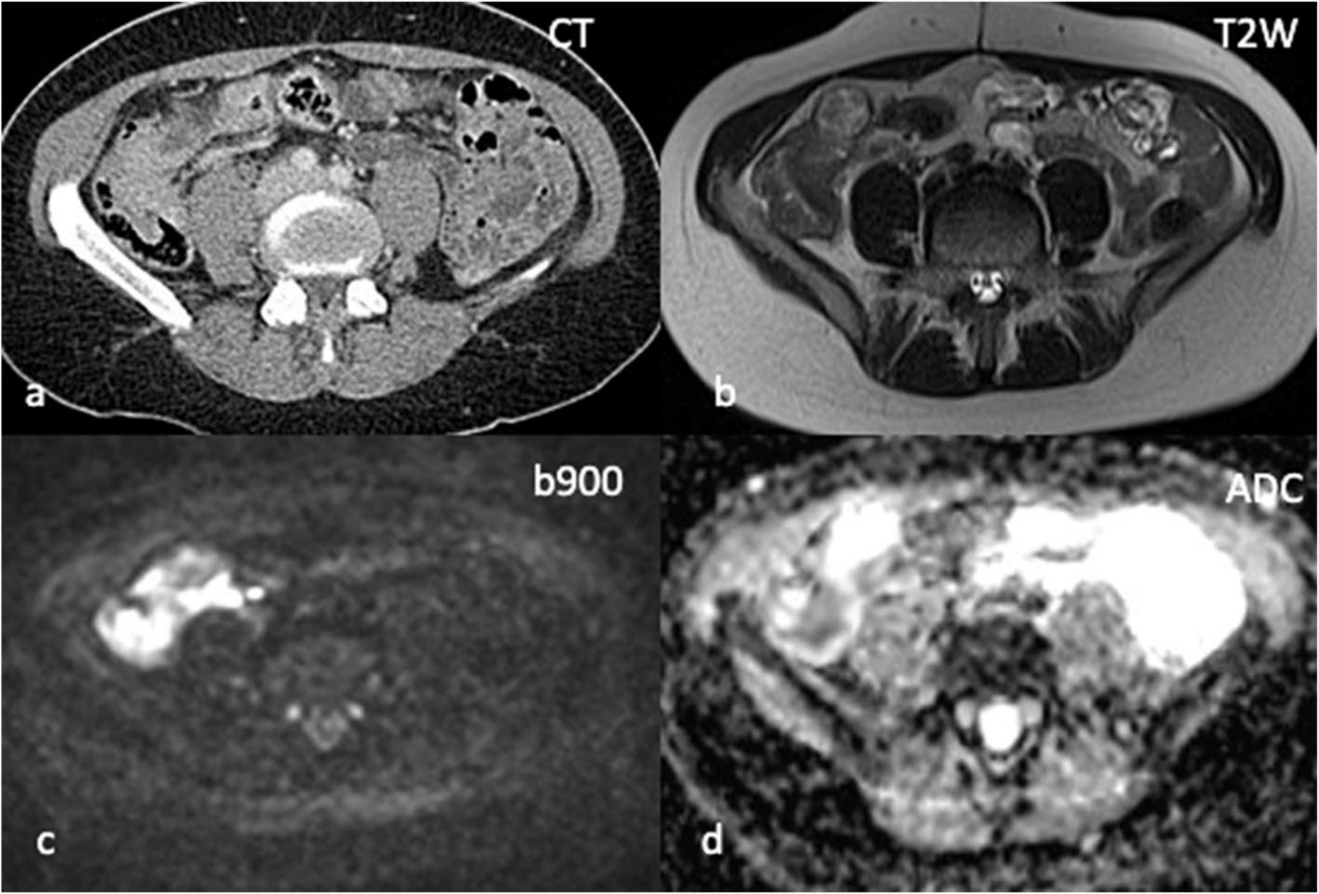
Peritoneal and serosal disease in 47-year-old female with BRCA 2 mutation and bilateral metastatic lobular breast cancer. No measurable peritoneal or serosal disease on axial CT (**a**). WB DWI-MRI performed within 2 weeks demonstrates thickening of the right peritoneal reflection on axial T2W sequence (**b**), restricted diffusion along the caecum and appendix on the b900 sequence (**c**) and corresponding low ADC values (**d**) in keeping with peritoneal and serosal metastatic disease

The more recently established DWI techniques have become invaluable in the assessment of peritoneal disease (Fig. 5). Cianci et al. evaluated the sensitivity of DWI in combination with MRI in the detection of peritoneal carcinomatosis in 24 patients with gastrointestinal or gynecological malignancies and reported that DWI in combination with MRI increased the sensitivity and detection of peritoneal carcinomatosis compared to MRI alone [59]. In this setting, FDG-PET/CT has a limited role and its main use would be in the detection of unsuspected extraperitoneal involvement [60].

### Imaging of metastatic breast cancer during pregnancy

Staging during pregnancy is challenging. Imaging modalities need to be selected with caution to minimise foetal exposure to ionising radiation. Associated risks of ionising radiation include miscarriage, stillbirth, mental retardation, organ malformation and carcinogenesis [61]. Staging in pregnancy has traditionally been performed with chest radiography (with appropriate abdominal shielding), and ultrasound [61] (Fig. 1). To evaluate the bones, brain, liver and pelvis, MRI is the modality of choice [51]. Despite some evidence of its safety during pregnancy [62], it has been shown in animal models that radiofrequency electromagnetic fields used in MRI could pose a risk to the foetus at an early gestational age [62]. Due to the fact that Gadolinum is known to cross the placental barrier [63] and potential concerns related to foetal toxicity, current recommendations advise that its use should be avoided, particularly during the first trimester of pregnancy unless absolutely necessary [62, 63]. A recent study evaluated the application of WB-MRI for staging in 14 pregnant patients in whom breast cancer was diagnosed during the second or third trimester [64]. Median gestational age at MRI was 20 weeks (range 13–32) and median gestational age at delivery was 36 weeks (range 32–38). No physical abnormalities were identified in any of the neonates. One case of respiratory distress syndrome and a further case of perinatal jaundice were described. There were no neonatal deaths. A solitary bone metastasis was reported in 1 patient and was confirmed on follow-up MRI. WB-MRI seems therefore feasible, accurate and safe in the second and third trimester of pregnancy [64]. Another recent study evaluated the feasibility of WB-MRI in 20 pregnant patients with suspected malignancy [65]. WB-MRI was performed in addition to routine staging procedures (diagnostic clinical/laboratory, surgical and imaging work-up including chest radiography, CT thorax, ultrasound and MRI). Among the patients, 10 had breast cancer, 3 Hodgkin’s lymphoma, 2 cervical cancer, 1 ovarian borderline tumor, 2 colon cancers, 1 lung cancer and 1 malignant conjunctival tumor. Of these, 8 were upstaged following WB-MRI. No adverse foetal effects were attributed to WB-MRI in this study. The authors followed that WB-MRI is feasible and safe during pregnancy and offers superior sensitivity and specificity to conventional staging imaging while reducing the need for multimodal imaging in this cohort. WB-MRI has several additional advantages including lack of contrast administration or use of ionising radiation and ability to accurately stage using a non-invasive, single-step imaging tool, possibly reducing diagnostic delays. However, more clinical data is needed to assess the safety, sensitivity and specificity of this technique during pregnancy.

## Novel drugs and response-assessment techniques

### Whole-body diffusion-weighted imaging

**Fig. 6:**
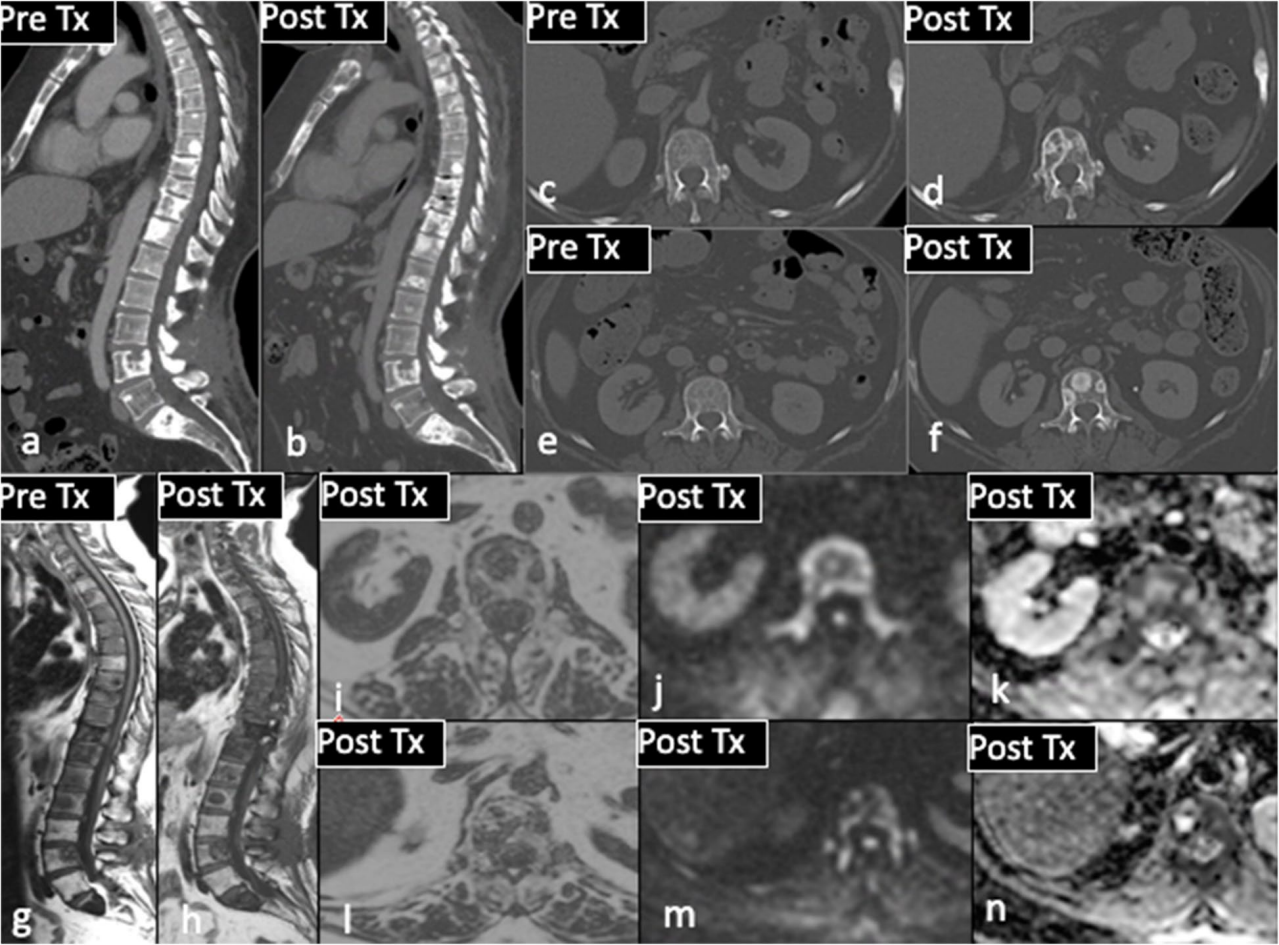
56-year-old female with grade 2 ER positive HER 2 negative invasive ductal carcinoma of the right breast: comparison of pre and post treatment CT (**a**-**f**) showing ‘new sclerotic ‘bone metastases in T10 and L1. Pre and post treatment T1W sagittal MRI sequences (**g**, **h**) show apparently new sclerotic foci (occult on CT). Post treatment WB DWI-MRI unequivocally proves treatment response, with low signal on axial fat fraction imaging (**i**, **l**), high b900 signal (**j**, **m**) and high ADC values (**k**, **n**) at the sites of previously identified disease

DW-MRI is an emerging tool in the scope of medical oncology. WB DWI-MRI is an attractive technique enabling early detection of skeletal metastases as well as spread to other sites (liver and brain) [66]. The diffusion-weighted sequence is acquired with a fat suppressed, free breathing technique, reducing the duration of the examination and the presence of artifacts [50]. DW-MRI measures the Brownian motion of water molecules within intra- and extracellular spaces. This occurs in highly cellular lesions or in environments in which tissue architecture is disrupted and can be quantified by calculating the apparent diffusion coefficient (ADC) [67]. Early signs of disease response or progression can be detected by early changes in water diffusivity [68]. Treatment response can be evaluated by changes on DW sequences, although the precise timing of diffusional changes remains disputed. Specifically, an increase in ADC value can precede macroscopic or radiological response of tumor cells to systemic chemotherapy [69] (Fig. 6). A retrospective study was conducted in 101 patients who underwent WB-MRI within 14 days of CT thorax, abdomen and pelvis examination [68]. Data on distribution, extent of disease and systemic anticancer therapy response assessment on WB DW-MRI and CT were compared. WB DW-MRI identified additional sites of disease in 53.3% of cases compared to CT. A difference in treatment decision was reported in 28% of cases, most commonly due to disease progression identified on DW-MRI. In 18.9% of these cases, stable disease was reported on CT [68]. Theilmann et al. monitored 13 patients with metastatic breast cancer and 60 measurable liver lesions with DW-MRI after initiation of new systemic chemotherapy [69]. In tumors responsive to treatment at 6 weeks, significant changes in ADC values were observed as early as day 4 and 11. DW-MRI could also have a role in evaluating tumor response in the skeleton where osteoblastic disease is considered unmeasurable according to RECIST 1.1. Given its sensitivity to bone marrow cellularity, the relative proportion of fat and marrow cells, water content and bone marrow perfusion, DW-MRI is playing an increasingly important role in the staging of metastatic bone disease [67]. As a result of advances in software application, ADC can be analysed by histograms allowing quantification of tumor volume. Histogram analysis permits quantitative and qualitative evaluation of changes in ADC values over time during treatment at metastatic sites within bone. This technique has been used by Padhani et al. to assess tumor response in the skeleton [70]. Tumor response can be divided into three ADC tracks: highly probable response (response with tissue necrosis), likely response (probably related to microscopic cellular necrosis) and absence of response (viable tumoral tissue) [67, 70]. Although WB-MRI is very sensitive, its specificity is poor when compared to FDG-PET/CT. In a study of 33 breast cancer patients with suspected disease recurrence, FDG-PET/CT was compared to WB-MRI. Tumor recurrence was found in 20 patients with 186 malignant foci detected on FDG-PET/CT and WB-MRI. A higher number of lymph nodes were detected by FDG-PET/CT while WB-MRI more accurately detected distant metastases. Sensitivity was 93% for WB-MRI and 91% for FDG-PET/CT; specificity was 86% and 90%, respectively [56]. Furthermore, to date WB-MRI is less widely available than FDG-PET/CT due to limited MRI scanning capacity, longer scanning times and the requirement to train radiologists in its interpretation.

### Contrast Clearance Analysis (CCA)

**Fig. 7:**
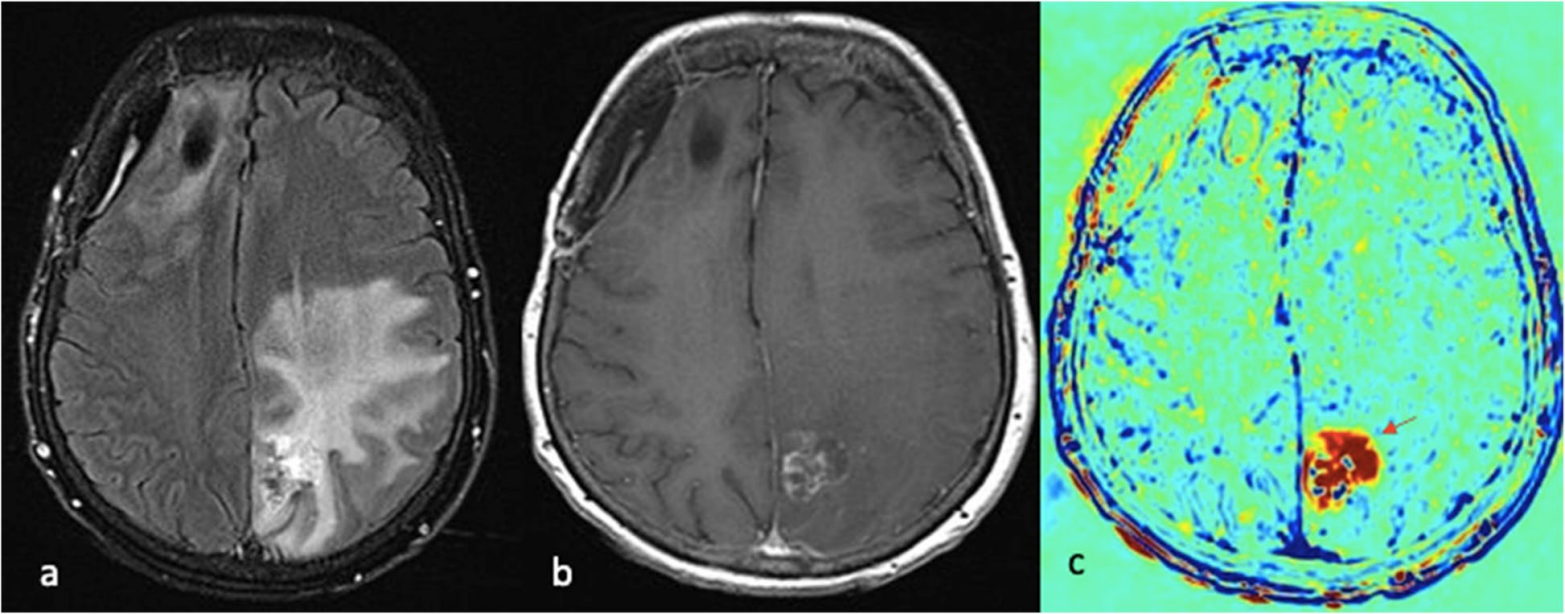
65-year-old patient with HER2 positive invasive ductal carcinoma of the right breast with metastases to the brain, lung and bone. Resected right frontal lobe metastasis and left parietal lobe deposit recently treated with stereotactic radiotherapy. Axial post-contrast FLAIR sequence (**a**) demonstrates confluent signal abnormality within the left parietal lobe. Axial post-contrast T1W sequence (**b**) shows heterogenous enhancement at the site of treated metastasis in the left partial lobe. CCA (**c**) shows corresponding contrast accumulation (red arrow) at the site of contrast enhancement, unequivocal for radiation necrosis rather than persistent tumoral activity

Persistent tumoral activity within the CNS can be indistinguishable from treatment-related effects on conventional imaging. The sequelae of radiation therapy in particular poses a diagnostic dilemma for radiologists. CCA, formerly termed Treatment Response Assessment Maps (TRAMs) is a novel MRI-based tool that aims to address this challenge. CCA utilises three-dimensional contrast-enhanced T1-weighted imaging to calculate the difference between early and delayed contrast clearance (5 and 60–105 min, respectively) to distinguish active tumor from treatment-related effects or pseudoprogression [71] (Fig. 7). Difference in contrast clearance at these two time points is used to create a colour map where *‘blue’* depicts regions with high contrast clearance due to *‘breakdown of the blood-brain barrier’* (high vascular activity related to active disease) and *‘red’*, to represent sites with low contrast clearance due to an *‘intact blood-brain barrier’* (low vascular activity in non-tumoral tissues) [71, 72]. Peker et al. compared CCA to standard clinicoradiology follow-up in 37 patients with a total of 130 intracranial metastases and reported a sensitivity of 96.1% and positive predictive value of 99.2% for CCA in determining radiation-effects [72]. Furthermore, CCA showed 2-fold sensitivity in diagnosing persistent tumoral activity compared to conventional T1- and T2-weighted MRI sequences. Despite their inherently lengthy acquisition times, CCA therefore holds huge promise in the accurate evaluation of treatment response in metastatic breast cancer involving the CNS.

### Hybrid imaging

PET/MRI is a novel hybrid technique combining the functional data of PET with the structural and anatomo-functional information acquired on MRI [73, 74]. PET/MRI significantly reduces exposure to ionising radiation while maintaining high-quality morphological information. In addition, this imaging technique overcomes a number of limitations of FDG-PET/CT and has been shown to outperform both conventional imaging and FDG-PET/CT in the detection of metastases at initial staging [73, 74]. In their recent study of 154 patients with newly diagnosed breast cancer, Bruckmann et al. demonstrated the superiority of FDG-PET/MRI compared to CT and BS; correctly detecting all bone lesions at initial staging [75]. In another study comparing FDG-PET/MRI to FDG-PET/CT in 51 patients with breast cancer, 30 of which had distant metastases, FDG-PET/MRI was found to be more sensitive for liver and possibly bone metastases, whereas FDG-PET/CT remained superior in the detection of lung metastases [73]. This is supported by Riola-Parada et al. in their systematic review comparing PET/MRI and FDG-PET/CT which showed a similar diagnostic performance between the two modalities with the exception of small lung metastases, in which FDG-PET-CT was superior [76]. FDG-PET/MRI is also superior in distinguishing benign from malignant lesions [74] and is performed at about half the radiation dose of FDG-PET/CT. However, FDG-PET/CT acquisition is faster, susceptible to less motion artifact and cheaper than FDG-PET/MRI. As such, further large-scale studies are needed to assess if the additional cost associated with FDG-PET/MRI is justifiable; improving overall outcomes and decision-making in patient care.

### Novel PET radiotracers

In patients with metastatic breast cancer treated with endocrine therapy, metabolic response assessed on FDG-PET/CT has been shown to be predictive of PFS [77]. Although FDG is the only validated tracer for treatment response assessment in metastatic breast cancer, it has some limitations. For example, FDG has minimal uptake in low grade tumors, is unable to differentiate inflammation from malignancy, has high physiological uptake in the brain and bowel and is less sensitive in lobular breast cancer subtypes (Fig. 5). New PET radiotracers are under investigation in preclinical or clinical studies. The Zephir trial evaluated tumor intra- and interpatient heterogeneity in HER2 mapping for patients with HER2-positive metastatic breast cancer. The investigators used ^89^Zr-Trastuzumab PET/CT and found positive lesions in 6 patients out of the 20 included [78]. This enables evaluation of tumor heterogeneity and therefore may predict response to targeted therapy. Several other radiotracers have been investigated; targets and examples include: proliferation (^18^ F-fluorothymidine [^18^ F-FLT]), tumor hypoxia (^64^Cu-diacetyl-*bis*N [4]-methyl thiosemicarbazone [^64^Cu-ATSM] and ^18^ F-fluoromisonidazole [^18^ F-FMISO], angiogenesis (Arg-Gly- Asp (R-G-D) based radiotracers targeting integrin α_v_β_3_ which is associated with angiogenesis [^18^ F-RGD]) and tumor endocrine receptor (ER) expression (the estrogen receptor with ^16^α-^18^ F-fluoroestradiol [^18^ F-FES]). ^18^ F-FES has been evaluated in several single centre studies with overall outcomes demonstrating good correlation between ^18^ F-FES uptake and ER expression level [79]. Moreover, other data suggest FES/PET could provide additional information over and beyond conventional clinical criteria to help predict response to endocrine therapy. In an exploratory study including 56 patients treated with Palbociclib and aromatase inhibitors for ER-positive metastatic breast cancer, patients underwent a ^18^ F-FES-PET/CT before treatment initiation. Nine of 10 patients with progressive disease had a ^18^ F-FES-negative site with a median PFS of 2.4 months [80]. Only 4 of 46 patients with ^18^ F-FES-positive lesions developed progressive disease with a median PFS of 23.6 months. For patients with ^18^ F-FES-positive lesions only, the median PFS was even longer (26.5 months versus 16.5 months). This could therefore help to better select ER-positive HER2-negative metastatic breast cancers who would derive benefit from Palbociclib in combination with endocrine therapy. Several clinical trials are ongoing to ascertain its true benefit (NCT03768479, NCT02398773, NCT02409316, NCT03442504, NCT01916122). If novel radiolabelled molecules are able to assess ER and HER2 expression with PET/CT, this could prove a helpful adjunct in guiding patient management without the need for repeat biopsy [81]. However, more research is required to evaluate this in the context of the new HER2-low subtype.

### Genomics and ctDNA detection

Major advances have been made in technical and analytic development, allowing better understanding of breast cancer biology. Recent advances in sequencing technologies have enabled the emergence of massively parallel sequencing techniques that allow comprehensive profiling of the entire genome of a cancer (all coding genes or a selection of genes). These DNA sequencing approaches offer the potential to deliver targeted therapy matching unique molecular alterations within a given tumor.

In addition, tissue biopsy and imaging do not provide sufficient information regarding real-time representation of disease biology, monitoring and tracking sensitive or resistance mechanisms. Tumor cells actively release circulating tumor DNA (ctDNA), cell-free DNA (cfDNA), microRNAs, non-coding RNA and microvesicles as a result of their spread both as single cells or clusters [82]. ctDNA can be detected in the plasma and serum of patients diagnosed with advanced cancer, with high levels associated with more aggressive phenotypes [83]. ctDNA could be used as a potential non-invasive tool to characterise the somatic genetic features of cancer cells [84]. Studies have demonstrated that ctDNA could be used to monitor tumor dynamics in various solid cancers [85]. A prospective study compared the sensitivity of ctDNA, CA15-3 and circulating tumor cells to CT in monitoring the disease burden in patients undergoing treatment for metastatic breast cancer [74]. Using targeted or whole-genome sequencing, 30 of 52 patients recruited were found to have genomic alterations suitable for monitoring. ctDNA was detected in 29 patients, whereas CA15-3 and circulating tumor cells were detected in 21 and 26 patients, respectively. ctDNA levels showed greater dynamic range and greater correlation with changes in tumor burden [84]. ctDNA dynamics could anticipate imaging-based disease progression [84]. For example, ctDNA dynamic analysis was performed in the PALOMA3 study [85]. A reduction in PI3KCA ctDNA levels at day 15 of Fulvestrant and Palbociclib therapy strongly predicted PFS (Hazard ratio 3.94, 95% CI 1.61–9.64, *p* = 0.0013) [86]. This could help to predict non-efficacy of treatment ahead of morphological changes on conventional imaging and avoid unnecessary treatment toxicity. A small prospective study included 49 ER-positive locally advanced or metastatic breast cancer patients and compared PI3KCA quantification with CEA, CA15-3 and CT assessments. The ctDNA dynamics, although often concordant with other parameters, did not always reflect tumor assessment on CT imaging, suggesting that ctDNA dynamics should be evaluated together with radiographic imaging [87].

Clonal heterogeneity of metastatic breast cancer can limit efficacy of targeted therapies. It can be evaluated by repeated biopsy at different time points and from different regions. However, this is an invasive procedure not without risks. ctDNA could be an interesting alternative as it may represent tumor spatial heterogeneity and could help detect oncogenic drivers [84]. The plasmaMATCH trial used mutations identified in ctDNA to select the appropriate treatment: extended-dose Fulvestrant for patients with ESR1 mutations, Neratinib for HER2 mutations, Capivasertib for AKT1 or PTEN mutations. This trial confirms the feasibility of ctDNA testing to select mutation-directed therapies in metastatic breast cancer [88]. Recently, Alpelisib, a PI3Kα-specific inhibitor was licensed in PI3KCA-mutated, ER-positive advanced breast cancer [89]. PI3KCA-mutated cancers can be detected on ctDNA to select the appropriate patients for Alpelisib therapy. As secondary resistance is responsible for approximately 80% of deaths in cancer patients, liquid biopsy could also be used to detect genomic alterations responsible for secondary resistance [90]. For example, the ESR1 mutation can be detected in up to 30% of tumors previously treated with aromatase inhibitors [91]. Preclinical studies suggest that these mutations result in cancer cells becoming insensitive to aromatase inhibitors but only partially resistant to endocrine therapies that target the ER directly [91]. Thus, detecting secondary mutations could guide oncologists in selecting the most appropriate therapy and further personalisation of appropriate imaging assessment. More data is still required to determine how this may be integrated into daily practise however. The randomised, open-label, phase III PADA1 trial included 1017 patients with ER-positive, HER2-negative metastatic breast cancer [92]. Among them, 279 developed a rising ESR1 mutation and 172 were randomly assigned to continuing Palbociclib and aromatase inhibitor (*n* = 84) versus switching to Fulvestrant and Palbociclib (*n* = 88) without evidence of disease progression on conventional imaging. Median PFS was significantly higher in the Fulvestrant group: 11.9 months versus 5.7 months (Hazard ratio 0·61, CI 0·43−0·86; *p* = 0·0040). ctDNA monitoring together with conventional imaging could therefore help to tackle acquired resistance and improve patient outcomes [91]. Other clinical trials, including the SERENA6 trial (NCT04964934), are ongoing and will try to address this question and confirm its clinical utility.

### Radiogenomics in metastatic breast cancer

Artificial intelligence (AI), the process of creating machines to simulate human thinking and behavior, is fast altering the horizon of medical imaging. Radiomics is the computerized analysis of medical images, extracting quantitative data and utilizing algorithms that can identify patterns within images, exploiting them to make predictions and assist with clinical decision-making [93, 94]. Deep learning is a particular subset of machine learning that utilizes multilayered neural networks with weighted connections between neurons that iteratively adjust through repeated exposure to raw training data [95]. Zhou et al. assessed the feasibility of deep learning applications to predict the likelihood of axillary lymph node metastases from ultrasound images acquired from primary breast cancers [96]. The best of three performing models yielded satisfactory predictions on the test set with an area under the receiver operating characteristic curve, sensitivity and specificity of 0.90, 82% and 79%, respectively. Moreover, the model outperformed three experienced radiologists. Basavanhally et al. have also shown that radiomics could help detect lymphocyte infiltration in HER2-positive breast cancers [97]. Similarly, the Memorial Sloan Kettering Cancer group evaluated the performance of AI and radiomics in assessing breast cancer receptor status and molecular subtypes from multiparametric MRI [98]. The authors concluded that their radiomic signatures were able to discriminate between treatment-naïve molecular breast cancer subtypes with high accuracy.

Radiogenomics combines genomic and radiomic imaging profiles to correlate imaging with gene expressions/mutations. Radiogenomics has the potential of improving knowledge of tumor biology and management of patients at the bedside. Fan et al. reported their radiogenomics signature was able to identify tumor heterogeneity and had a prognostic value [99]. However, it is well documented that machine learning is affected by selection bias. This is to say that while algorithms frequently demonstrate diagnostic accuracy similar to or exceeding that of radiologists when presented with known training or test set images, performance is often less impressive when algorithms are presented with new, real-world images. This is often made more challenging by the ‘black box’ phenomenon - the idea that there is uncertainty which data is being used by the algorithm to facilitate a diagnosis [100]. Despite this, radiogenomics could in the future play an important role in diagnosis, staging, prognostication, disease monitoring and predicting treatment response in metastatic breast cancer.

## Multimodal disease assessment in metastatic breast cancer

Multimodal imaging where two or more techniques are combined, is not a new concept in the setting of metastatic breast cancer. In perhaps its most common form, the combination of more than two modalities in the staging of metastatic disease is now commonplace in many institutions. In fact, a recent study conducted by the author’s group at the Royal Marsden Hospital, found that the application of multimodal imaging does indeed influence real-world decision-making in the management of metastatic breast cancer. Specifically, the authors reported additional sites of disease, earlier recognition of progressive disease and changes to systemic chemotherapy when WB-MRI was paired with CT, BS or FDG-PET/CT [101].

Increasingly the term ‘multimodal’ is more recently being applied to the realm of evolving hybrid imaging techniques, such as the association of PET with MRI. With the evolution and advancement of more novel functional and molecular diagnostic tools, as well as AI however, this idea is arguably now outdated and warrants reappraisal.

Interpretation of multimodal ‘data’, rather than multimodal ‘imaging’, is likely to become increasingly important in the delivery of accurate and timely diagnosis and management of patients with metastatic breast cancer. This said, the expansion of multimodal data assessment is often costly and timely. In their retrospective study of 1307 breast cancer patients undergoing surgery, Ojala et al. reported that primary surgery was significantly affected by requirement for additional imaging or other diagnostic tests [102]. Thus, while the evolution of multimodal and multiparametric imaging and data analysis will inevitably continue, it must not come at the cost of timely and quality cancer care which maximises patient safety and improves overall patient outcome.

## Conclusion

Major progress has been made in oncological imaging over the last few decades. Accurate disease assessment at diagnosis and during treatment is important in the management of metastatic breast cancer, in which CT (and BS if appropriate) is generally widely available, relatively cheap and sufficient in many cases. However, several additional imaging modalities are emerging and can be used as adjuncts, particularly in pregnancy or other diagnostically challenging cases of metastatic breast cancer (e.g. lobular subtypes, inflammatory cancers). Nevertheless, no single imaging technique is without limitation. The authors have evaluated the vast array of imaging techniques – individual, combined parametric and multimodal - that are available or that are emerging in the management of metastatic breast cancer. This includes WB DW-MRI, CCA, novel PET breast cancer-epitope specific radiotracers and radiogenomics. This ongoing oncologic-imaging evolution will help to provide more accurate diagnosis and treatment response evaluation and represents an opportunity to develop personalised medicine protocols that improve management and overall outcomes for patients with metastatic breast cancer.

### Original points

- The strengths and limitations of metastatic breast cancer anatomical and functional imaging techniques are described.
- Contrast Clearance Analysis (CCA) is described as a new MRI technique useful for differentiating *‘active tumor’* from *‘benign change & inactive treated residua’* in CNS metastases.
- Multimodal research directions including novel PET radiotracers, radiogenomics, ctDNA are presented.
- WB-MRI is likely to play a role in staging of lobular breast carcinoma and in the response assessment of bone-only/bone-predominant disease and infiltrative liver disease in the presence of pseudocirrhosis.

## Supplementary Information

**Additional file 1.**

## Data Availability

Not applicable

## Abbreviations

CT: Computer tomography
MRI: Magnetic resonance imaging
PET: Positron emission tomography
PET/CT: Positron emission tomography/CT
FDG-PET/CT: 2-deoxy-2 [^18^ F] fluoro-D-glucose PET/CT
BS: Bone scintigraphy
WB-MRI: Whole-body MRI
DWI: Diffusion-weighted imaging
ADC: Apparent diffusion coefficient
WB DW-MRI: Whole-body DW-MRI
PET/MRI: Positron emission tomography/MRI
CCA: Contrast Clearance Analysis
ctDNA: Circulating tumor DNA
SPECT: Single Photon Emission Computed Tomography
CT-SPECT: CT-Single Photon Emission Computed Tomography
