# Supplementary material for: Imaging in metastatic breast cancer, CT, PET/CT, MRI, WB-DWI, CCA: review and new perspectives
Source: Cancer Imaging. 2023 May 31;23:53. doi: 10.1186/s40644-023-00557-8 (PMC10230813; doi:10.1186/s40644-023-00557-8)
Supplement: Supplementary file 1 — Additional file 1. [file 40644_2023_557_MOESM1_ESM.pdf]

Editor

Cancer Imaging

23rd March 2023

Dear Editor,

Manuscript title: **Imaging in Metastatic Breast Cancer, CT, PET/CT, MRI, WB-DWI, CCA: Review and New Perspectives**

We would like to again thank both the reviewers for their time and prudent comments. We have made further alterations which we hope address the points raised. We have strived to provide additional detail where requested and removed content that does not contribute towards the overall narrative. Where alterations have not been made we hope that the explanations provided are satisfactory and clarify our thinking.

Reviewer 2 (Comments to the Author):

The article addressing an interesting topic. Clearly a lot of work has gone in to preparing the manuscript and the revisions. Apologies, there is no intension of being contrarian but aspects still come across as slightly random regarding structure and content:

1. The section from line 165 onwards relates to MRI (without mentioning the modality) in breast screening - is this within the scope of an article on imaging in metastatic breast cancer - if so, should are other modalities such as full field digital mammography, digital breast tomosynthesis, contrast enhanced spectral mammography and ultrasound (grey scale, elastography etc) be incorporated or could this section be truncated?

   Thank you for your comments. We have implemented your suggestion and truncated this paragraph to remove mention of breast screening and provide a more cohesive narrative.

2. The topic transfers from breast MRI to whole body MRI mentioning diffusion weighted imaging (DWI). The use of DWI in other body systems is alluded to subsequently and not until after that are basic principles of DWI outlined in a section 4 - the order is perhaps not ideal.

   Thank you for the above comments. The introductory section on MRI has been truncated and now references the subsequent dedicated section where this is expanded upon in detail. The subsequent section has also been re-titled to improve clarity for the reader. We hope this improves the flow of the paper.

3. Optical imaging related to breast screening is discussed - is this within the scope?

   Thank you for your comment. We appreciate this may distract from the focus of the paper and have removed this paragraph.

4. Lines 281-282 Why is experience of the image reported specifically highlighted regarding CT interpretation and liver metastases? "It is… the experience of the reporting radiologist influences performance in detection and assessment of liver metastases on CT". As imaging, particularly oncologic becomes more and more subspecialised could it be argued that this is the case with most cross-sectional imaging tests and specific disease types/sites? If the sentence is retained, please could a page be added to the reference - apologies I could not find the origin of the statement.

   Thank you highlighting this point. The comment has been subsequently removed.

5. In this section (Lines 283-304) there is more detail on CT and yet it is correctly argued that MRI is more sensitive and specific with minimal comment. It may be worth adding some more discussion here.

   Thank you for your comment. We agree with your comment and have added further discussion on MRI utility in this context. Given the wider clinical availability of CT however, we have retained the discussion of CT assessment of hepatic disease.

6. The highly relevant aspect of breast subtypes is alluded to with a section specifically on lobular cancer and peritoneal carcinomatosis. Is there particular reason this type and anatomical site is highlighted as opposed to other areas? Due to its non-mass spreading nature lobular cancer metastasis may be notoriously difficult to detect and subsequently follow in many body sites; on FDG PETCT they may not be particularly avid.

   Thank you for your comment. This section has been expanded to recognise other challenging sites of metastatic disease in ILC. We have focussed particularly on peritoneal metastases in ILC as we consider this a common challenge in breast imaging. We feel this provides a good example to discuss the relative merits of DWI imaging and limitations of conventional CT and FDG-PET/CT.

7. Section 5 Lines 578-596. These studies relate to assessing neoadjuvant chemotherapy in primary disease - as above - is this within the stated scope of the article?

   Thank you for your comment. The highlighted comments referencing assessment of neoadjuvant chemotherapy have been removed. Further comment has been added to reflect the scope of the article.

Thank you again for your careful consideration of our manuscript. We hope that we have addressed all the points raised. We would be very happy to make any further changes and we would welcome any further comments.

Yours sincerely,

Dr Bhupinder Sharma, FRCR Consultant, The Royal Marsden Hospital, London Senior Lecturer, Honorary Faculty, The Institute of Cancer Research, London Expert Advisor to UK NICE Centre for Guidelines Development (CfG)
